# Gemcitabine-Based Bladder Preservation in BCG-Unresponsive High-Risk NMIBC: Evidence, Limitations, and Clinical Positioning

**DOI:** 10.3390/biomedicines14081821

**Published:** 2026-08-13

**Authors:** Aris Kaltsas, Konstantinos Papathanasiou, Ilias Giannakodimos, Athanasios Zachariou, Nguyen Phuc Cam Hoang, Mai Ba Tien Dung, Tran Vinh Hung, Michael Chrisofos, Nikolaos Sofikitis, Fotios Dimitriadis

**Affiliations:** 1 Third Department of Urology, Attikon University Hospital, School of Medicine, National and Kapodistrian University of Athens, 12462 Athens, Greece; akaltsas@med.uoa.gr (A.K.); iliasgiannak@med.uoa.gr (I.G.); mchrysof@med.uoa.gr (M.C.); 2 First Department of Urology, Faculty of Medicine, School of Health Sciences, Aristotle University of Thessaloniki, 54124 Thessaloniki, Greece; papathanasioukonstantinos@hotmail.com; 3 Department of Urology, Faculty of Medicine, School of Health Sciences, University of Ioannina, 45110 Ioannina, Greece; zahariou@otenet.gr (A.Z.); nsofikit@uoi.gr (N.S.); 4 Departments of Andrology and Urology, Binh Dan Hospital, Ho Chi Minh City 70000, Vietnam; npchoang@gmail.com (N.P.C.H.); maibatiendung@yahoo.com (M.B.T.D.); hungsurgeon2021@yahoo.com (T.V.H.)

**Keywords:** BCG-unresponsive, non-muscle-invasive bladder cancer, gemcitabine, docetaxel, TAR-200, INLEXZO, bladder preservation, radical cystectomy, patient-reported outcomes

## Abstract

Bacillus Calmette–Guérin (BCG)-unresponsive non-muscle-invasive bladder cancer (NMIBC) is a high-risk disease state for which early radical cystectomy remains the guideline-supported oncologic reference in surgically fit patients. Bladder-sparing therapy is necessary for patients ineligible for or declining cystectomy, but it is a preference-sensitive trade-off rather than an equivalent alternative: failure may permit high-grade recurrence, progression, and loss of a curative window. This targeted narrative review synthesizes the evidence for intravesical gemcitabine monotherapy, sequential gemcitabine–docetaxel, and the sustained-release gemcitabine intravesical system TAR-200/INLEXZO, updated through 4 August 2026. Because the review is not systematic and the evidence is dominated by single-arm and retrospective studies, cross-study comparisons are descriptive and establish neither superiority nor equivalence; many gemcitabine studies enrolled mixed BCG-failure cohorts that do not satisfy the contemporary definition. Gemcitabine monotherapy is active but shows declining disease control over time. Sequential gemcitabine–docetaxel has accumulated substantial multicenter observational experience, yet a 2026 retrospective comparison did not demonstrate improved high-grade recurrence-free survival over gemcitabine alone. TAR-200 achieved a centrally confirmed complete response at any time in 82.4% of patients, with a median duration of response of 25.8 months in the single-arm phase 2b SunRISe-1 study and is approved in the United States as INLEXZO for BCG-unresponsive carcinoma in situ with or without papillary tumors; no approved agent holds a papillary-only indication. Comparative patient-reported outcome evidence remains limited, and molecular markers, urinary tumor DNA and transcriptomic subtypes remain investigational rather than validated selection tools. Bladder-sparing treatment should therefore be phenotype- and label-aware, time-limited, and coupled to intensive surveillance with predefined triggers for cystectomy.

## 1. Introduction

Bladder cancer is the ninth most commonly diagnosed cancer worldwide, with marked geographic and sex-based disparities in incidence and mortality [1,2]. In the United States, approximately 75% of newly diagnosed cases are non-muscle-invasive bladder cancer (NMIBC) [3], a biologically distinct disease state characterized by frequent recurrence and, in high-risk subsets, progression [2,4]. For high-risk NMIBC, transurethral resection followed by intravesical bacillus Calmette–Guérin (BCG) immunotherapy remains a cornerstone of management, but a substantial proportion of patients experience recurrence or become unresponsive to BCG, defining a therapeutically challenging disease state [3,4].

The term BCG-unresponsive NMIBC was developed to identify patients in whom further BCG is unlikely to provide durable disease control and to standardize eligibility criteria and endpoints for clinical trials of novel agents [5]. BCG-unresponsive high-risk NMIBC includes persistent or recurrent carcinoma in situ (CIS), with or without papillary disease, within 12 months after adequate BCG; recurrent high-grade Ta/T1 disease within 6 months after adequate BCG; or high-grade T1 disease at the first evaluation after induction BCG. Adequate BCG is defined here according to the current European Association of Urology (EAU) criteria as the completion of at least five of six doses of an initial induction course plus at least two of three doses of a second induction course, or two of three doses of maintenance therapy [6]. It should be noted that the U.S. Food and Drug Administration (FDA) and International Bladder Cancer Group (IBCG) formulation differs slightly in the second-induction criterion, which it expresses as at least two of six instillations of a second induction course [5,7]. The distinction is small but not trivial, because a patient who received two of six doses of re-induction satisfies the FDA wording while not necessarily satisfying the EAU wording, and would therefore be counted as BCG-unresponsive under one framework and as BCG-exposed under the other. We use the EAU criteria throughout and, where a study applied the FDA definition, we say so [4,6].

BCG intolerance is a separate disease state, caused by an inability to complete adequate BCG because of toxicity, and is not included within the BCG-unresponsive population. High-grade disease after inadequate BCG, and delayed high-grade recurrence after adequate BCG outside the BCG-unresponsive windows but within 24 months, fall within the BCG-exposed spectrum; recurrences beyond 24 months should be described separately as late relapse [6]. The historical labels “BCG-refractory”, “BCG-relapsing”, and “BCG-failure” therefore cannot be assumed to satisfy the contemporary regulatory definition, and cross-study comparisons that mix these categories may overstate efficacy in genuinely BCG-unresponsive cohorts [5,7]. The regulatory framework was further operationalized through FDA guidance for drug and biologic development, which emphasizes standardized eligibility, pathologic confirmation, risk stratification, and clinically meaningful endpoints such as complete response and duration of response in CIS-containing cohorts [7].

High-risk NMIBC that persists or recurs after adequate BCG is consequential because the goals of treatment extend beyond visible tumor clearance to durable prevention of high-grade recurrence, progression, metastasis, and disease-specific mortality [3]. Early radical cystectomy remains the guideline-supported oncologic standard for surgically fit patients with BCG-unresponsive high-risk NMIBC [3,4].

The rationale for bladder-sparing therapy is to avoid or defer major surgery and urinary diversion and, where possible, to preserve native urinary, sexual, and psychosocial function in patients who are medically ineligible for radical cystectomy or who decline it after informed shared decision-making. Radical cystectomy is associated with substantial perioperative morbidity, and standardized reporting series demonstrate clinically important early complication rates as well as durable functional and quality-of-life implications [8]; in a contemporary prospective cohort of patients facing this same decision, cystectomy carried a 90-day mortality of 2.5% and a higher burden of adverse and serious adverse events than bladder-sparing therapy [9]. This is, however, a preference-sensitive trade-off rather than an assumption of oncologic or quality-of-life equivalence. Repeated transurethral resections, intravesical or systemic treatment, cystoscopy, cytology, biopsy, and uncertainty about recurrence themselves impose urinary symptoms, functional impairment, anxiety, time burden, and financial toxicity.

The potential oncologic cost of bladder preservation must be stated explicitly. Failure of a bladder-sparing strategy can permit persistent CIS or recurrent high-grade disease to progress, and may narrow the window for curative surgery, particularly when several successive salvage regimens are administered. In a retrospective FDA-defined BCG-unresponsive cohort of 149 patients, of whom 60 underwent initial cystectomy and 89 initial bladder-sparing therapy, patients undergoing cystectomy after two or more bladder-sparing lines had ≥pT3 or node-positive disease in 42% of cases, compared with 18% after one bladder-sparing line and 15% after upfront cystectomy (*p* = 0.038); the individual subgroups are small and the comparison is non-randomized [10]. In a 10-site cohort of 578 patients meeting FDA BCG-unresponsive criteria, initial bladder-sparing therapy was not associated with statistically different metastasis-free, cancer-specific or overall survival compared with upfront cystectomy; within the same cohort, however, node-positive disease was found in 13% of patients undergoing delayed cystectomy after bladder-sparing therapy compared with 4% after upfront cystectomy, and the 5-year risk of bladder cancer death rose with successive lines of bladder-sparing therapy, reaching 28% among patients who received three lines [11]. Both readings of that study are given here deliberately: the overall survival comparison is reassuring, whereas the pathology and mortality gradients across successive salvage lines are not, and a balanced account requires both. These retrospective findings are subject to treatment-selection bias and do not establish that delayed cystectomy caused the adverse pathology; nevertheless, they support a time-limited treatment strategy with predefined triggers for surgery rather than indefinite sequential salvage. An intact bladder should therefore not be used as a surrogate for either oncologic safety or quality of life, and every bladder-sparing plan should incorporate prespecified response assessments and an explicit exit strategy for timely cystectomy [3,4,12].

Interest in intravesical chemotherapy has been further intensified by recurrent worldwide BCG shortages, which have constrained access to first-line therapy and renewed attention to chemotherapeutic alternatives and salvage regimens [13]. Among these, intravesical gemcitabine is attractive because it is a deoxycytidine analogue with established antitumor activity in urothelial carcinoma and a pharmacologic profile that permits local administration with limited systemic exposure [14,15]. Conventional intravesical administration is nonetheless limited by the short dwell time of single instillations and by urinary washout, so strategies that increase exposure time or combine agents with non-overlapping mechanisms have been explored to improve efficacy without substantially increasing toxicity [15,16,17]. The principal gemcitabine-based approaches are gemcitabine monotherapy, sequential gemcitabine–docetaxel, and TAR-200, an intravesical drug-releasing system designed to provide sustained gemcitabine delivery over several weeks [15,16,17].

The clinical question is not whether these therapies can produce responses—several regimens have demonstrated measurable activity—but whether they can produce responses durable enough to justify delaying or avoiding radical cystectomy in carefully selected patients [15,17,18,19]. Durability is particularly important for CIS, where high early complete response rates may not translate into sustained high-grade recurrence-free survival or long-term bladder preservation [17,20,21].

This is a narrative rather than a systematic review. The underlying evidence is dominated by single-arm trials and retrospective cohorts with heterogeneous definitions of BCG failure, disease phenotypes, surveillance schedules, endpoint timing, and response ascertainment. Study identification, selection, and weighting were not governed by a registered protocol, a Preferred Reporting Items for Systematic Reviews and Meta-Analyses (PRISMA)-based study flow, duplicate screening, formal risk-of-bias assessment, or Grading of Recommendations, Assessment, Development and Evaluations (GRADE) certainty grading. This review therefore provides a clinically oriented map of the evidence, but it cannot assign formal certainty ratings or support causal rankings among therapies; comparisons drawn across separate studies are descriptive only and should not be interpreted as evidence of superiority or equivalence.

## 2. Scope and Methods of the Narrative Review

Consistent with the structural limitation stated in Section 1, no PRISMA-based study flow, duplicate screening, formal risk-of-bias assessment or certainty grading was undertaken. A targeted narrative framework was chosen because the relevant evidence is heterogeneous, spanning monotherapy, sequential chemotherapy, continuous-release device therapy, and comparator bladder-sparing agents that have not been compared head to head. The search and source-selection process was investigator-directed and may therefore be susceptible to selection, reporting, and publication bias. PubMed and the Cochrane Library were searched through 4 August 2026 for peer-reviewed clinical evidence using combinations of terms related to bladder cancer, NMIBC, BCG-unresponsive disease, gemcitabine, docetaxel, TAR-200, intravesical chemotherapy, and bladder-sparing therapy; the full search strings for each database, with the record counts they returned, are provided in Supplementary Table S1. Embase was not available to the authors and was therefore not searched. No language restriction was applied at the search stage, but non-English records were excluded at screening. Both restrictions are stated again among the limitations in Section 9.6. Because guideline and regulatory documents are not consistently captured by bibliographic databases, guideline documents and FDA regulatory sources were checked separately through 4 August 2026. Additional articles were identified through manual screening of reference lists and major urologic and oncologic congress proceedings. Conference proceedings were used only to identify emerging studies and contextual developments; numerical efficacy and safety outcomes were taken from peer-reviewed publications or primary regulatory documents.

Priority was given to prospective clinical trials, large multi-institutional series, comparative studies, guideline statements, regulatory documents, and meta-analyses. The outcomes of greatest interest were complete response, recurrence-free survival, high-grade recurrence-free survival, duration of response, cystectomy-free survival, bladder preservation, cancer-specific survival, adverse events, treatment discontinuation, patient-reported outcomes, and follow-up duration. Evidence was synthesized qualitatively because the included studies differ substantially in population, BCG-failure definition, histologic composition, inclusion of CIS, dosing schedule, maintenance strategy, endpoint definition, and follow-up duration. No new pooled estimates, *p* values, or comparative effect estimates were calculated; numerical outcomes are reported as published in the primary sources.

For each clinical study we recorded the original BCG-failure label, prior BCG exposure, timing of recurrence, and disease phenotype, and classified applicability as follows: (i) strict contemporary BCG-unresponsive, used only for cohorts requiring adequate BCG plus CIS recurrence within 12 months, high-grade Ta/T1 recurrence within 6 months, or high-grade T1 disease at the first evaluation after induction; (ii) BCG-exposed, for cohorts that included BCG intolerance, recurrence after a single induction course, or delayed high-grade recurrence outside the BCG-unresponsive windows but within 24 months; (iii) late relapse, for recurrence beyond 24 months; and (iv) historical, pre-definition BCG-refractory or BCG-relapsing cohorts, or cohorts with unspecified timing. Where a study did not report the interval from the last BCG dose with enough precision to separate categories (ii) and (iii), this is stated rather than assumed. Study design was additionally recorded as prospective single-arm, randomized, comparative observational, retrospective multi-institutional, or regulatory. BCG-naïve studies were excluded from the principal efficacy synthesis and are cited only for contextual discussion. This study-level applicability audit is presented in Supplementary Table S2. These classifications were used to describe clinical applicability and did not constitute a formal risk-of-bias or certainty-of-evidence assessment. Descriptions such as “more extensively studied” or “more mature evidence base” refer to the volume and duration of the available evidence, and not to formally graded certainty or to comparative efficacy.

Because regulatory and publication status for several therapies remains time-sensitive, statements about approval or availability reflect information available as of 4 August 2026.

## 3. Pharmacologic Rationale for Gemcitabine in the Bladder

Gemcitabine (2′,2′-difluoro-2′-deoxycytidine, dFdC) is a deoxycytidine analogue that requires intracellular phosphorylation to active metabolites; it inhibits ribonucleotide reductase, depletes the deoxynucleotide pools required for DNA synthesis, and is incorporated into DNA, leading to masked chain termination that is relatively resistant to DNA repair [14]. These properties make gemcitabine S-phase-dependent and underscore the importance of adequate intracellular exposure time, which is directly relevant to intravesical delivery.

In the bladder, the urothelial permeability barrier and short physiological dwell time limit the duration of drug–tumor contact, and continuous urine production progressively dilutes and washes out instilled agents. This pharmacologic constraint motivates the three gemcitabine-based strategies discussed below: optimizing conventional instillation (monotherapy), combining gemcitabine with a second cytotoxic agent that has a complementary mechanism (sequential gemcitabine–docetaxel), and engineering sustained local release to extend exposure time well beyond a single dwell (TAR-200) [14,16,17]. Prolonged exposure is pharmacologically plausible because gemcitabine activity is exposure-time dependent, but direct claims of superior tissue penetration or tumor-cell kill should be considered hypothesis-generating unless supported by pharmacokinetic or tissue-level studies [14,17].

Docetaxel is a taxane that promotes microtubule assembly, stabilizes microtubules by inhibiting depolymerization, and interferes with mitotic progression [22]. The rationale for combining it with gemcitabine rests on the use of agents with non-overlapping mechanisms and toxicities; however, a direct claim that docetaxel overcomes gemcitabine resistance, or that the specific sequence is mechanistically superior in human bladder tissue, is not established by the available clinical data.

## 4. Gemcitabine Monotherapy

### 4.1. Historical and Mixed BCG-Failure Evidence

Early studies of intravesical gemcitabine predated, or did not consistently apply, the contemporary BCG-unresponsive definition. Their results remain informative regarding antitumor activity and feasibility, but they should not be interpreted as direct estimates of efficacy in a strictly defined contemporary registration population.

In the 2006 Memorial Sloan Kettering phase II study, the title used the term BCG-refractory, but eligibility also permitted BCG-intolerant patients; the cohort therefore predates, and cannot be considered equivalent to, a strictly defined contemporary BCG-unresponsive population. Among 30 patients, who received gemcitabine 2 g twice weekly for 3 weeks with a repeat course after a rest interval, intravesical gemcitabine achieved a complete response rate of 50% at 8 weeks, but the median recurrence-free survival was 3.6 months and the 1-year recurrence-free survival among responders was approximately 21%, indicating that early response did not reliably translate into durable disease control [15].

SWOG S0353 evaluated gemcitabine 2 g weekly for 6 weeks followed by monthly maintenance for up to 12 months in patients who had recurred after at least two prior BCG courses; of 58 patients enrolled, 47 were evaluable for response, of whom 47% were disease-free at 3 months, with recurrence-free survival decreasing to 28% at 1 year and 21% at 2 years [18]. The majority of this high-risk cohort had CIS, the histology most relevant to contemporary BCG-unresponsive registration trials. SWOG S0353 required recurrence after at least two prior BCG courses but permitted the last BCG treatment up to 3 years before registration; strict timing-based BCG-unresponsive eligibility therefore cannot be assumed.

The randomized Di Lorenzo trial suggested lower recurrence with gemcitabine than repeat BCG after initial BCG failure (52.5% vs. 87.5%) and higher 2-year recurrence-free survival (19% vs. 3%), but this population—failure after a single BCG induction course—does not correspond to the modern strict BCG-unresponsive category, and adequate BCG exposure was not established [23]. A Cochrane review concluded that intravesical gemcitabine may have favorable effects on recurrence compared with some intravesical comparators, including mitomycin C and, in a small high-risk study, BCG, but the certainty of evidence was low or very low for several comparisons [24]. This distinction matters because meta-analyses across broad NMIBC populations include treatment-naive, recurrent, BCG-exposed, and BCG-failure cohorts that may not reflect outcomes in the highest-risk group [5,24].

A subsequent single-arm, open-label study enrolled 36 patients defined by contemporary BCG-unresponsive or BCG-intolerant criteria who were unwilling to undergo radical cystectomy; intravesical gemcitabine (weekly induction followed by monthly maintenance for 12 months) yielded disease-free survival of 68.8% after induction and 44.4% and 31.7% at 12 and 24 months, respectively, with a median follow-up of 27 months and no treatment-related deaths [25]. Because BCG-intolerant patients were also included, this was not a purely BCG-unresponsive cohort; it therefore reinforces the feasibility of gemcitabine monotherapy as a bladder-preserving salvage option without altering the conclusion that durability is limited.

### 4.2. Contemporary Study-Defined and Retrospectively Reclassified Evidence

More recent studies have either prospectively applied, or retrospectively reconstructed, contemporary BCG-unresponsive criteria, and therefore provide more directly applicable—although still predominantly non-randomized—evidence.

In a Korean multicenter retrospective series of 131 patients with BCG-unresponsive high-risk NMIBC (41.9% with CIS) who were unable or unwilling to undergo radical cystectomy, intravesical gemcitabine achieved 1- and 2-year recurrence-free survival of 68% and 42% and 1- and 2-year progression-free survival of 87% and 77%, with 2-year cancer-specific and overall survival of 98% and 97%, over a median follow-up of 25 months; the regimen was well tolerated, with only 5.3% of patients unable to complete induction [26]. This was a contemporary, study-defined BCG-unresponsive cohort treated after adequate BCG; however, early relapse was defined as high-grade recurrence within 1 year for all phenotypes, so full concordance with the FDA 6-month window for papillary Ta/T1 disease cannot be confirmed.

A 2025 Memorial Sloan Kettering re-analysis of single-agent intravesical gemcitabine after BCG failure reported similar 12-month recurrence-free survival across historical BCG-refractory and contemporary BCG-unresponsive definitions and identified positive pretreatment urine cytology as a marker associated with recurrence and progression, supporting the need for improved minimal residual disease assessment before salvage therapy [27]. Of the 127 patients analyzed, 57% met the historical definition of BCG-refractory disease and 33% met BCG-unresponsive criteria using a 12-month cut-off, so the overall estimates should not be generalized to a strictly defined population.

From a practical standpoint, gemcitabine monotherapy is commonly administered at 1 to 2 g in 50 to 100 mL normal saline on a weekly induction schedule for 6 weeks, with institution-dependent maintenance [15,18]. Tolerability is favorable, although grade-specific toxicity is not uniformly reported across these datasets. Applying a consistent reporting order where the sources permit it: in SWOG S0353 the toxicity-evaluable population was 55 patients, with grade 3 toxicity in 3 of 55 (5.5%), no grade 4 or 5 events, and one discontinuation, which followed a grade 2 event rather than a severe one [18]; in the contemporary Korean series 5.3% of 131 patients did not complete induction [26]; elsewhere, adverse events are described only qualitatively as local lower urinary tract symptoms such as urgency, frequency and dysuria, and no grade ≥ 3 treatment-related rate is reported [15,24].

Taken together, these studies demonstrate clinically relevant activity and favorable local tolerability, but disease control declines over time, and the evidence does not support characterizing gemcitabine monotherapy as a reliably durable strategy in strictly defined BCG-unresponsive disease.

## 5. Sequential Gemcitabine–Docetaxel

### 5.1. Foundational and Mixed BCG-Exposed Cohorts

The sequential gemcitabine–docetaxel regimen was first described in 2015, when an initial single-institution experience in 45 patients—treated between 2009 and 2014, partly motivated by BCG supply shortages—established the dual-instillation schedule of six weekly gemcitabine (1 g) instillations followed immediately by docetaxel (37.5 mg); treatment success was 66% at first surveillance, 54% at 1 year, and 34% at 2 years over a median follow-up of 15 months [28]. This cohort included BCG-naïve, BCG-intolerant and single-course-failure patients, so it establishes the regimen rather than its efficacy in BCG-unresponsive disease, and its outcome figures are reported here for that purpose only. This foundational report introduced the dosing concept later refined in the larger multi-institutional and long-term series discussed below.

Sequential intravesical gemcitabine–docetaxel is commonly administered as gemcitabine 1 g in 50 to 100 mL with a 60 to 90 min dwell, followed by bladder drainage and immediate instillation of docetaxel 37.5 mg with a 60 to 120 min dwell, usually weekly for 6 weeks, with monthly maintenance for 12 to 24 months in disease-free patients in many institutional protocols [16].

In a multi-institutional series of 276 patients with recurrent NMIBC after BCG exposure, sequential gemcitabine–docetaxel produced 1- and 2-year high-grade recurrence-free survival rates of 65% and 52%, respectively, with 3.6% progressing on transurethral resection and no patient-, disease-, or treatment-related factors associated with failure [16]. This was not a pure BCG-unresponsive cohort: only 105 patients (38%) met contemporary BCG-unresponsive criteria, and 31 patients had low-grade tumors, so the overall 1- and 2-year estimates are mixed-population estimates and should not be generalized to strictly defined high-risk BCG-unresponsive disease [16,29]. Tolerability in that series was reported descriptively rather than by Common Terminology Criteria for Adverse Events (CTCAE) grade: 26 patients had symptoms that affected the treatment schedule and 9 patients (3.3%) were unable to tolerate a full induction course [16,29].

Long-term follow-up from the University of Iowa cohort (97 patients, 71% with CIS-containing disease, 35% meeting the BCG-unresponsive definition) reported a 3-month complete response rate of 74%, a median duration of response of 25 months, 5-year high-grade recurrence-free survival of 30%, 5-year cystectomy-free survival of 75%, and 5-year cancer-specific survival of 91% [19]. These data support the concept that a meaningful subset of patients can maintain bladder preservation after sequential gemcitabine–docetaxel, although the non-randomized design and the mixed eligibility of the cohort limit definitive conclusions [19].

A European multicenter cohort collected data on 95 patients treated at 12 centers, with outcomes reported for 75 patients; 1-year disease-free survival was 73%, high-grade disease-free survival 79%, and progression-free survival 95% after gemcitabine–docetaxel in high- and very-high-risk NMIBC previously treated with BCG [30]. Eligibility required only a completed BCG induction course and at least one follow-up evaluation, without a requirement for adequate BCG or a recurrence-timing window; this cohort is therefore best described as BCG-exposed rather than BCG-unresponsive, and the short follow-up and protocol variability further limit interpretation.

### 5.2. Evidence in Contemporary BCG-Unresponsive Disease

In a separate multicenter series restricted to 102 patients with BCG-unresponsive disease, 6-, 12-, and 24-month high-grade recurrence-free survival was 78%, 65%, and 49%, respectively; six patients progressed to muscle-invasive disease, and BCG-refractory disease was associated with a higher risk of high-grade recurrence than BCG-relapsing disease (hazard ratio [HR] 2.14; 95% confidence interval [CI] 1.02–4.49) [31].

In a multicenter analysis restricted to BCG-unresponsive patients (gemcitabine–docetaxel, *n* = 95; additional BCG, *n* = 204), sequential gemcitabine–docetaxel was associated with better progression-free survival, cystectomy-free survival, and cancer-specific survival than additional BCG, whereas high-grade recurrence-free survival, metastasis-free survival, and overall survival were similar between groups [32]. Because this evidence is observational, these findings should be interpreted as associations rather than proof of causal superiority. This comparison also addressed additional BCG rather than approved bladder-sparing therapies or radical cystectomy, and residual confounding by indication cannot be excluded.

In a prospective cohort of 39 patients with recurrent high-grade NMIBC treated with gemcitabine–docetaxel, 28 of 39 enrolled patients (72%) met BCG-unresponsive criteria and 31 (79%) had CIS; among the 37 patients evaluable for efficacy, 1-year high-grade disease-free survival was 67% overall and 73% in the BCG-unresponsive subgroup, although sample size and follow-up were limited [33].

### 5.3. Contribution of Docetaxel and the Influence of BCG-Failure Phenotype

The incremental contribution of docetaxel to intravesical gemcitabine has now been examined directly. A 2026 retrospective comparative-effectiveness study evaluated 296 patients with high-risk NMIBC treated at three sites with gemcitabine monotherapy (*n* = 173) or sequential gemcitabine–docetaxel (*n* = 123). Median high-grade recurrence-free survival was 22.6 months with gemcitabine and 19.5 months with gemcitabine–docetaxel, and the doublet was not associated with improved high-grade recurrence-free survival after adjustment (adjusted HR 1.09; 95% CI 0.65–1.82; *p* = 0.80). No advantage was demonstrated in the BCG-unresponsive sensitivity analysis (*n* = 89; HR 2.24; 95% CI 0.96–5.24; *p* = 0.06), and among the 49 patients with BCG-unresponsive CIS, median high-grade recurrence-free survival was 12.2 months with gemcitabine and 7.6 months with the doublet (*p* = 0.12). Grade ≥ 3 adverse events were rare in both groups (2.3% vs. 2.4%). Imbalances in maintenance utilization (19% vs. 40%), baseline characteristics, gemcitabine dosing, and follow-up duration, together with the limited size of the BCG-unresponsive subgroup, preclude a conclusion of equivalence. These findings nevertheless challenge the assumption that adding docetaxel necessarily improves oncologic outcomes, and they reinforce that combination-specific or sequence-specific superiority remains unproven [34].

The efficacy of gemcitabine–docetaxel may be further refined by the pattern of prior BCG failure. In a multicenter series of 143 patients stratified by FDA/IBCG-defined failure phenotype (72 early non-refractory, 36 early refractory, and 35 late BCG-relapsing), 12-month recurrence-free survival was highest in late BCG-relapsing disease (84%; 95% CI 70–100), intermediate in early non-refractory failure (67%; 95% CI 55–82), and lowest in early refractory failure (54%; 95% CI 36–82), with parallel differences in high-grade recurrence-free survival, whereas 12-month progression-free survival exceeded 90% across all groups [35]. On multivariable analysis, both early non-refractory (adjusted HR 3.20) and early refractory failure (adjusted HR 3.59) carried a higher recurrence risk than late relapse; confidence intervals for these adjusted estimates are not reported in the source, so the precision of the ranking cannot be assessed. Importantly, the late BCG-relapsing subgroup falls outside the strict BCG-unresponsive time window. Under the current EAU categories it belongs either to the BCG-exposed spectrum, if recurrence occurred within 24 months, or to late relapse, if it occurred beyond that point; the published report does not resolve this split, so the subgroup should be read as non-BCG-unresponsive evidence rather than assigned to a single category [6]. These data indicate that gemcitabine–docetaxel retains meaningful activity across BCG-failure phenotypes, and that the failure pattern should inform expected durability and counseling rather than the choice of agent.

Observed clinical heterogeneity is at present better explained by the pattern of BCG failure than by any molecular selection assay. The association between BCG-refractory disease and higher high-grade recurrence (HR 2.14; 95% CI 1.02–4.49) is prognostically informative and can guide counseling and surveillance intensity, but it does not establish that a molecularly defined subgroup should preferentially receive gemcitabine–docetaxel rather than another bladder-sparing option [31,35].

Tolerability is a relative strength of the regimen, although it is reported inconsistently. In the prospective cohort of 39 patients, adverse events were recorded without causality attribution: any event occurred in 30 of 39 (77%), a single grade 3 event occurred (pyelonephritis requiring hospitalization, 1 of 39, 2.6%) after which treatment was successfully resumed, and 3 of 39 (7.7%) discontinued because of side effects [33]. In the retrospective comparison with gemcitabine monotherapy, grade ≥ 3 events occurred in 2.4% of 123 patients receiving the doublet and 2.3% of 173 receiving gemcitabine alone [34]. The largest multicenter series did not report CTCAE-graded toxicity; 26 patients had symptoms that affected the treatment schedule and 9 of 276 (3.3%) could not complete induction [16,29]. Comparative experience with BCG reports lower rates of dysuria and hematuria, but without graded denominators [16,32]. Taken together, sequential gemcitabine–docetaxel has accumulated substantial multicenter and long-term observational experience, but its evidence base remains predominantly retrospective, the only published prospective cohort was small and non-randomized, and its incremental benefit over gemcitabine monotherapy has not been demonstrated. It should therefore be presented as a commonly used off-label bladder-sparing option supported by encouraging but non-definitive evidence, and not as a superior or preferred regimen [16,19,32,34].

## 6. Continuous Intravesical Gemcitabine Delivery: TAR-200/INLEXZO

TAR-200 is an intravesical drug-releasing system designed to provide sustained local delivery of gemcitabine. It is inserted through a co-packaged urinary catheter using a dedicated stylet by a trained healthcare professional, remains in the bladder for a 3-week indwelling period, and is subsequently removed; cystoscopic assistance may be required for retrieval but is not required for routine insertion [17,36]. The clinical premise is that prolonged local exposure may overcome the rapid washout that limits conventional instillation, consistent with the S-phase-dependent, exposure-time-sensitive pharmacology of gemcitabine [14,17]; claims of enhanced tissue penetration or superior tumor-cell kill should be presented as hypotheses unless directly supported by pharmacokinetic or tissue-level data.

In the phase 2b SunRISe-1 study, TAR-200 monotherapy in the CIS cohort (*n* = 85) achieved a centrally confirmed complete response rate of 82.4% and a median duration of response of 25.8 months [17]. In the papillary disease-only cohort (*n* = 52), 12-month disease-free survival was 70.2% [17]. In the CIS cohort, grade ≥ 3 treatment-related adverse events occurred in 12.9%, serious treatment-related adverse events in 5.9%, and discontinuation due to treatment-related adverse events in 3.5%, with no deaths attributed by investigators to treatment (the regulatory label, which applies different attribution rules to the same 85-patient population, records a fatal adverse reaction rate of 1.2%); in the papillary-only cohort the corresponding figures were 13.5%, 5.8%, and 7.7% [17]. The investigators reported that TAR-200 monotherapy showed the most favorable balance of activity and tolerability among the SunRISe-1 cohorts; the comparator cohorts are not reported here in equivalent detail, so this within-study observation should not be read as a formal comparison [17].

The SunRISe-1 efficacy and durability findings are clinically notable, but the study was single-arm. In particular, a complete response ascertained at any time is not interchangeable with a complete response assessed at a fixed 3-month time point, so the SunRISe-1 figures should be read against their own assessment schedule. The distinctive current role of TAR-200 is its on-label sustained intravesical delivery for BCG-unresponsive CIS with or without papillary tumors, balanced against device logistics and the need for prompt cystectomy at failure. Activity in the papillary-only cohort is clinically relevant but falls outside the current U.S. indication [17,37].

On 9 September 2025, the FDA approved the gemcitabine intravesical system, INLEXZO (formerly TAR-200), for adults with BCG-unresponsive NMIBC with CIS, with or without papillary tumors. Because regulatory status is time-sensitive and this statement is based on an FDA announcement rather than a PubMed-indexed trial report, it is cited separately from the peer-reviewed SunRISe-1 publication [37].

## 7. Comparator Bladder-Sparing Therapies

Several bladder-sparing therapies have received U.S. regulatory approval for BCG-unresponsive NMIBC with CIS, with or without papillary tumors. Pembrolizumab was approved by the FDA in January 2020 for patients who are ineligible for or elect not to undergo cystectomy [38]. Nadofaragene firadenovec-vncg (Adstiladrin) was approved in December 2022 [39]. Nogapendekin alfa inbakicept-pmln (N-803; Anktiva) plus BCG was approved in April 2024 [40]. INLEXZO was approved on 9 September 2025 [37].

### 7.1. Systemic Immune Checkpoint Inhibitors

In KEYNOTE-057 Cohort A, pembrolizumab, a programmed cell death protein-1 (PD-1) inhibitor, achieved a 3-month complete response rate of 41%; the median duration of complete response was 16.2 months, and 46% of complete responders maintained complete response for at least 12 months [20]. Among the 101 patients in the Cohort A safety analysis, grade 3–4 treatment-related adverse events occurred in 13%, serious treatment-related adverse events in 8%, and treatment-related adverse events led to discontinuation in 7%, with no treatment-related deaths [20]. Immune-mediated toxicity is a relevant consideration because systemic immunotherapy has a toxicity profile distinct from local intravesical chemotherapy.

In the separately reported papillary-only cohort (Cohort B) of KEYNOTE-057, which enrolled 132 patients with BCG-unresponsive high-grade Ta or T1 disease without CIS, pembrolizumab produced a 12-month disease-free survival of 43.5% (95% CI 34.9–51.9); grade 3–4 treatment-related adverse events occurred in 14% and serious treatment-related adverse events in 13%, with no treatment-related deaths [41]. Cohort A and Cohort B safety data are reported separately here because the two cohorts differ in population and exposure and should not be combined. KEYNOTE-057 Cohort B provides papillary-only contextual efficacy data, but the regulatory indication for pembrolizumab in NMIBC remains CIS-containing BCG-unresponsive disease, with or without papillary tumors [38]; accordingly, FDA guidance recommends randomized, time-to-event trial designs for papillary-only BCG-unresponsive disease rather than direct extrapolation of CIS endpoints [7].

Not all systemic checkpoint inhibitors have shown comparable results. In the single-arm phase 2 SWOG S1605 trial of intravenous atezolizumab for BCG-unresponsive high-risk NMIBC, the CIS complete response rate at 6 months was 27% and the 18-month event-free survival for Ta/T1 disease was 49%, but the study did not meet its prespecified efficacy threshold. In the safety population of 166 patients, grade 3–4 treatment-related adverse events occurred in 23 (14%) and grade 3–5 treatment-related adverse events in 26 (16%), including three treatment-related deaths (immune-related myasthenia gravis with respiratory failure, immune-related myositis, and sepsis) [42]. Atezolizumab is not approved for this indication and is noted here only as context, underscoring that systemic immune-checkpoint inhibition carries a distinct risk profile and is not uniformly effective in BCG-unresponsive disease.

### 7.2. Intravesical Gene- and Cytokine-Based Therapies

Nadofaragene firadenovec (Adstiladrin) is a non-replicating adenoviral vector delivering the interferon alfa-2b gene, administered intravesically. In the pivotal phase 3 trial, nadofaragene firadenovec achieved a 3-month complete response rate of 53.4% in patients with BCG-unresponsive CIS with or without high-grade Ta/T1 disease, with 45.5% of complete responders maintaining response at 12 months [21]. In the FDA review population, the complete response rate was 51%, with a median duration of response of 9.7 months [39]. Five-year follow-up showed that bladder preservation was maintained in approximately half of treated patients [43]. Among the 157 patients in the safety population, study-drug-related grade 3–4 adverse events occurred in 6 patients (4%) and study-drug-related serious adverse events in 3 patients (1.9%); three patients discontinued because of adverse events, and there were no treatment-related deaths. Serious events considered procedure-related rather than study-drug-related are counted separately in the source and are not included in that figure, and the all-cause grade 3–4 rate in the same population was 18% (29 of 157) [21]. Patients with upper-tract disease, prostatic urethral involvement, lymphovascular invasion, micropapillary disease, or hydronephrosis were excluded, which is relevant when generalizing to unselected practice [21].

In QUILT-3.032, N-803 (nogapendekin alfa inbakicept; Anktiva) plus BCG produced a complete response in 58 of 82 patients with BCG-unresponsive CIS with or without papillary disease, corresponding to a complete response rate of 71%, with a median duration of response of 26.6 months [44]. In the FDA approval summary, the reported complete response rate was 62% in the regulatory efficacy population, with 58% and 40% of complete responders maintaining response for at least 12 and 24 months, respectively [40]. Safety was characterized in 88 patients using regulatory adverse-reaction definitions rather than investigator-attributed treatment-related adverse events: serious adverse reactions occurred in 16%, permanent discontinuation in 7%, and one fatal adverse reaction (1.1%) was reported; an aggregate grade ≥ 3 adverse-reaction rate is not reported in the label [40]. The primary publication reports a different quantity for a different population—treatment-emergent events in patients receiving BCG plus N-803—stating that most were grade 1–2 (86%) and that three grade 3 immune-related treatment-emergent events occurred; the two sets of figures are therefore not interchangeable [44].

Valrubicin remains a historical comparator for BCG-refractory CIS. In the pivotal cohort of 90 patients, a complete response confirmed by biopsy and cytology at 6 months was observed in 16 patients (18%), and 7 of 143 patients (5%) scheduled to receive six instillations did not complete them because of local bladder symptoms; its durability is limited and it should not be emphasized as a contemporary preferred option [45].

### 7.3. Regulatory Status, Treatment Selection, and Emerging Agents

Regulatory status is phenotype-specific. Current U.S. FDA indications for INLEXZO, nadofaragene firadenovec-vncg, and nogapendekin alfa inbakicept-pmln plus BCG are restricted to BCG-unresponsive NMIBC containing CIS, with or without papillary tumors. Pembrolizumab carries the same phenotype restriction and is indicated for patients who are ineligible for or have elected not to undergo cystectomy. None of these indications extends to papillary-only high-grade Ta/T1 disease, and sequential gemcitabine–docetaxel remains off-label in both phenotypes. Activity reported in papillary-only cohorts should therefore be described as evidence-supported off-label use rather than as an extension of an approved indication, and cross-study comparisons should distinguish complete response and duration of response in active CIS from disease-free or recurrence-free survival after complete resection of papillary-only disease [7,37,38,39,40].

**Figure 1:**
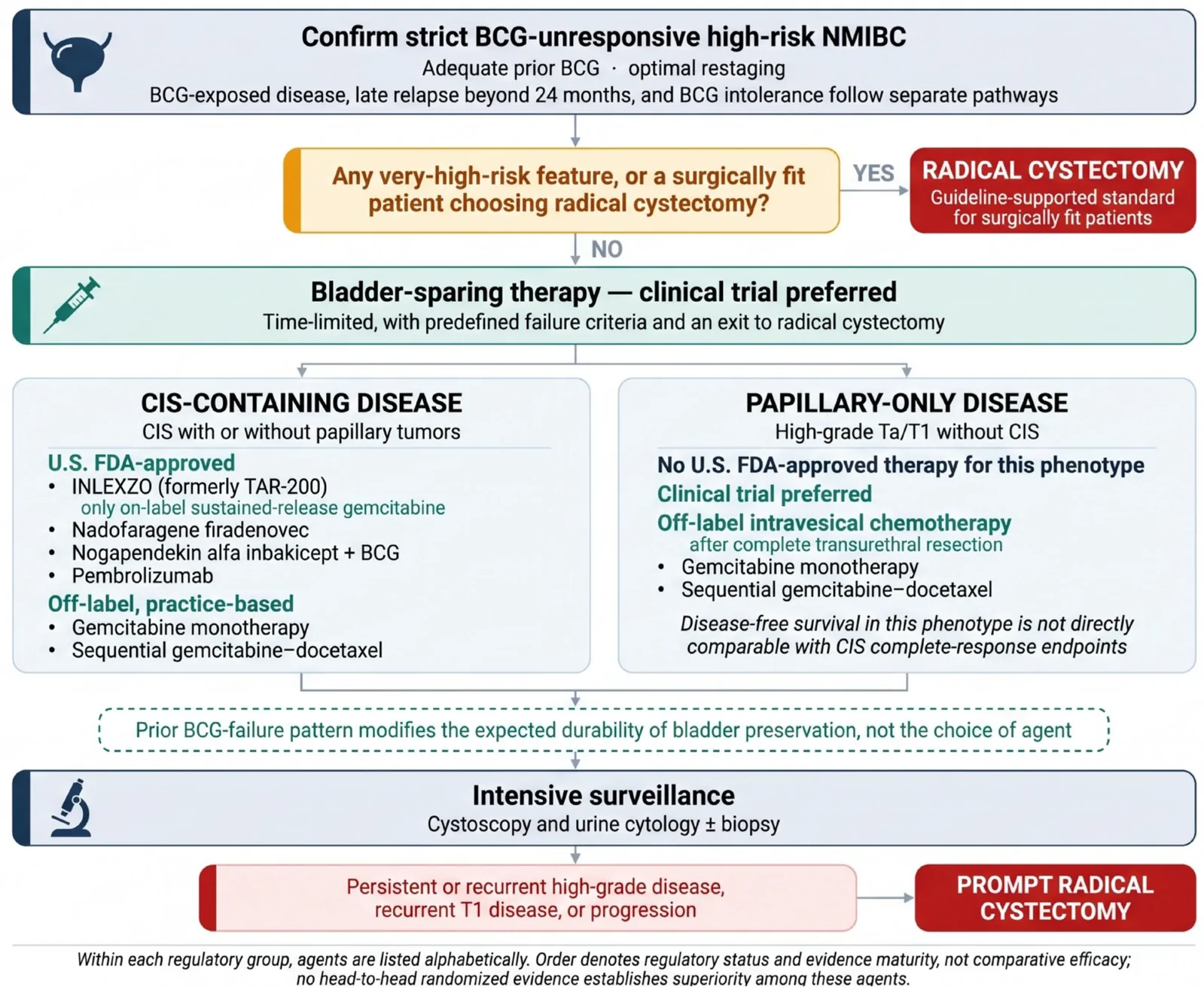
Risk-, phenotype-, and regulatory-status-stratified framework for BCG-unresponsive high-risk NMIBC. BCG-exposed disease (high-grade disease after inadequate BCG, or delayed high-grade recurrence after adequate BCG outside the BCG-unresponsive windows but within 24 months), late relapse beyond 24 months, and BCG intolerance are distinct states that follow separate pathways. Very-high-risk features comprise recurrent high-grade T1 disease, variant histology, lymphovascular invasion, prostatic urethral involvement, hydronephrosis or suspected upper-tract disease, and inability to comply with intensive surveillance. Within each regulatory group agents are listed alphabetically; ordering denotes regulatory status and evidence maturity, not comparative efficacy, and CIS complete-response and duration-of-response endpoints are not directly comparable with papillary-only disease-free survival. Investigational molecular biomarkers are deliberately excluded from the pathway because none is validated for treatment selection. Abbreviations: BCG, bacillus Calmette–Guérin; CIS, carcinoma in situ; NMIBC, non-muscle-invasive bladder cancer. Created in BioRender. Kaltsas, A. (2026) https://BioRender.com/m6a0z5y.

Because no randomized head-to-head trial has compared gemcitabine–docetaxel, TAR-200, pembrolizumab, nadofaragene firadenovec, or N-803 plus BCG, an evidence-based efficacy hierarchy cannot currently be established. Within the gemcitabine family, both single-agent gemcitabine and sequential gemcitabine–docetaxel have observational support; the doublet has accumulated more multicenter experience, but its incremental benefit over gemcitabine alone remains unproven and was not demonstrated in a recent retrospective comparative analysis [34]. TAR-200 reported a high response rate and durable responses in a single-arm phase 2b study and is the only sustained-release gemcitabine option with a regulatory indication [17,37]. Selection should therefore be driven by disease phenotype, regulatory indication, surgical fitness, toxicity profile, treatment burden, BCG availability, local expertise, access, and patient priorities, rather than by numerical cross-trial response ranking [3,4,12]. These principles are operationalized in the proposed clinical framework (Figure 1), which separates the options by regulatory status and evidence maturity rather than by efficacy.

Among the agents holding a U.S. FDA indication for CIS-containing disease, the practical discriminators are already contained in the evidence summarized above and can be applied in sequence: whether the patient can receive concomitant BCG at all, since nogapendekin alfa inbakicept is BCG-dependent and therefore shortage-sensitive; whether systemic immune-related toxicity is acceptable, which distinguishes pembrolizumab from the three intravesical options; whether the trial eligibility of a given agent matches the patient, since the nadofaragene firadenovec program excluded lymphovascular invasion, micropapillary histology, prostatic urethral involvement and upper-tract disease; and whether the service can support device insertion, a 3-week indwelling period and scheduled removal, which is specific to INLEXZO. The off-label, practice-based options apply when no approved option is available, accessible or acceptable. In papillary-only disease this separation does not apply, because no agent holds a U.S. indication for that phenotype; there, clinical-trial enrollment should be offered first, followed by complete transurethral resection with off-label intravesical chemotherapy, consistent with the phenotype-specific guideline recommendations for papillary Ta/T1 disease [3,6]. None of these discriminators is a claim of comparative efficacy, and none should be read as one.

The BCG-unresponsive NMIBC landscape is evolving beyond currently approved agents and gemcitabine-based strategies. The most advanced investigational bladder-sparing approaches are the oncolytic adenoviral immunotherapy cretostimogene grenadenorepvec (CG0070) and the non-viral gene-mediated therapy detalimogene voraplasmid (EG-70) [46,47]. In the single-arm, international phase 3 BOND-003 Cohort C study, intravesical cretostimogene grenadenorepvec achieved a centrally confirmed complete response at any time in 75.5% of evaluable patients with BCG-unresponsive CIS, with estimated 12- and 24-month duration-of-response rates of 64.2% and 60.1%, respectively, and no grade 3 or 4 treatment-related adverse events, treatment-related discontinuations, or treatment-related deaths [48]. In the phase 2 CORE-001 combination with pembrolizumab, the 12-month complete response rate was 57.1% with no progression to muscle-invasive disease [49]. Despite these clinically notable findings, cretostimogene remained investigational with no marketing approval for this indication as of 4 August 2026, and it should not be positioned as an approved treatment or ranked indirectly against approved agents. Sasanlimab plus BCG has also generated phase 3 data, but the CREST program addresses BCG-naïve high-risk NMIBC and is therefore adjacent contextual evidence rather than BCG-unresponsive salvage evidence [50,51]. Table 1 summarizes the population applicability, evidence design, key efficacy, standardized safety, and principal limitations of these gemcitabine-based and comparator bladder-sparing therapies.

## 8. Practical Considerations: Dosing, Patient Selection, and Surveillance

### 8.1. Patient Selection and Guideline Framework

Patient selection is central to the safe use of bladder-sparing therapy; the guideline framework itself is set out in the Introduction and in Section 9.1 and is not repeated here. The 2024 IBCG recommendations emphasize that bladder-sparing therapy in BCG-unresponsive NMIBC should be individualized according to patient selection, disease phenotype, treatment sequencing, and readiness to proceed to radical cystectomy at failure [12].

The 2026 EAU NMIBC guideline update reinforces this framework: it provides dedicated treatment recommendations for BCG-unresponsive disease—separately for papillary Ta/T1 tumors and for CIS with or without papillary tumors—and for the different categories of BCG failure, incorporating gemcitabine-based and device-based bladder-sparing options [6].

### 8.2. Dosing and Administration

For gemcitabine monotherapy, common dosing is 1 to 2 g in 50 to 100 mL normal saline weekly for 6 weeks, with variable maintenance [15,18,24]. For sequential gemcitabine–docetaxel, gemcitabine 1 g is followed by docetaxel 37.5 mg after bladder drainage, weekly for 6 weeks with monthly maintenance for 12 to 24 months in many series [16,19,32]. For TAR-200, the sustained-release system is placed intravesically using a co-packaged urinary catheter and stylet and removed at the end of each 3-week indwelling period [17].

Following FDA approval, the INLEXZO prescribing information specifies a 225 mg gemcitabine intravesical system placed once every 3 weeks for up to 6 months (up to 8 doses) during induction, followed by once every 12 weeks for up to 18 months (up to 6 doses) during maintenance, with removal of the system at the end of each 3-week indwelling period [36]. These practical parameters should be incorporated into scheduling and patient counseling and verified against the current label before use. The prescribing information also emphasizes practical safety constraints, including avoidance in patients with bladder perforation or compromised bladder mucosal integrity, magnetic resonance imaging (MRI)-conditional considerations, potential embryo–fetal toxicity, hazardous-drug handling requirements, and a warning that delayed cystectomy in patients with persistent CIS may increase the risk of muscle-invasive or metastatic disease [36].

### 8.3. Surveillance and Management of Failure

Surveillance after any bladder-sparing strategy must be intensive, because high-grade recurrence after salvage therapy may represent a narrow window before progression. Surveillance should follow high-risk NMIBC protocols, including early cystoscopy and cytology, biopsy when indicated—particularly in CIS—and prompt radical cystectomy for high-grade persistence, recurrent high-grade T1 disease, or progression in surgically fit patients [3,4]. The INLEXZO label reports that, among 83 evaluable patients with BCG-unresponsive CIS treated in Cohort 2 of SunRISe-1, seven patients (8%) progressed to muscle-invasive (T2 or greater) bladder cancer, with progression determined at the time of cystectomy in three patients (3.5% of the 85-patient safety population); this reinforces the need for prompt reassessment and cystectomy referral when persistent or recurrent high-grade disease is identified [36]. Variation in enhanced cystoscopy, imaging, and mandatory-biopsy schedules across BCG-unresponsive NMIBC trials can affect efficacy assessment, particularly in single-arm studies, supporting standardized surveillance and evaluation protocols when interpreting bladder-sparing data [52].

## 9. Discussion

The evidence supports the activity and feasibility of gemcitabine-based bladder-sparing therapy but does not establish a preferred regimen. Gemcitabine monotherapy is active and well tolerated but shows declining disease control over time in contemporary high-risk populations [15,18,24,26]. Sequential gemcitabine–docetaxel is supported primarily by retrospective multicenter cohorts and one small prospective series, and its incremental benefit over gemcitabine alone has not been demonstrated [16,19,32,34]. TAR-200 produced a high complete-response rate in a single-arm phase 2b study, but differences in eligibility, endpoint timing, assessment, and follow-up preclude direct comparison [17]. Each approach should therefore be interpreted within its own study design, phenotype, and regulatory context rather than ranked by unadjusted cross-trial response rates.

### 9.1. Clinical Positioning and Radical Cystectomy as the Oncologic Reference

A recent scoping review synthesizing evidence across BCG-naïve and BCG-unresponsive populations concluded that intravesical gemcitabine–docetaxel has transitioned from an experimental salvage approach towards mainstream bladder-sparing use, while emphasizing that the absence of randomized trials means it is not a formal standard of care [53]. This is consistent with the present positioning of gemcitabine–docetaxel as an increasingly adopted regimen supported mainly by non-randomized evidence, without implying equivalence or superiority to approved bladder-sparing agents.

The central clinical principle is that bladder preservation should not be conflated with oncologic equivalence to radical cystectomy in surgically fit patients. Radical cystectomy remains the guideline-supported standard for eligible patients, and bladder-sparing therapy should be used within a framework that prioritizes early detection of failure and timely surgery [3,4]. This is especially relevant for patients with CIS, multifocal high-grade disease, recurrent high-grade T1 disease, variant histology, lymphovascular invasion, prostatic urethral involvement, or suspected occult upper-tract disease—features incorporated into EAU risk stratification that argue for caution before prolonged bladder-sparing attempts [4].

Contemporary observational evidence nevertheless suggests that a closely monitored initial bladder-sparing attempt is feasible in selected patients. In a 10-site retrospective cohort of 578 patients meeting FDA BCG-unresponsive criteria, of whom 28% underwent upfront radical cystectomy and 72% received initial bladder-sparing therapy, bladder-sparing therapy was not associated with statistically different metastasis-free, cancer-specific, or overall survival over a median follow-up of 50 months. Within the bladder-sparing group, however, high-grade recurrence occurred in 37% and 52% of patients at 12 and 24 months, progression to muscle-invasive and/or metastatic disease in 7% at 12 months, 13% at 24 months and 19% at 48 months, and radical cystectomy was undertaken in 12% and 25% by 12 and 24 months. In total, 132 patients in that group (31.7% of the bladder-sparing cohort) ultimately underwent cystectomy, and second-, third- and fourth-line bladder-sparing therapy was given to 141 (33.9%), 54 (13.0%) and 24 (5.8%) patients, respectively; all percentages in this sentence are expressed relative to the bladder-sparing group rather than to the full cohort of 578 [11]. Because treatment was not randomized, these data support feasibility rather than oncologic equivalence, and the escalating recurrence, progression, and adverse pathology observed with successive salvage lines argue for protocol-defined assessment points, repeated counseling about cumulative risk, and an explicit threshold for timely cystectomy [10,11].

### 9.2. Heterogeneity, Endpoints, and Comparative Limitations

A central limitation of the evidence is heterogeneity. Studies differ in their definitions of BCG failure, and several influential gemcitabine studies predate the contemporary BCG-unresponsive category [5,15,18]. Cohorts also differ by inclusion of CIS, papillary-only disease, prior BCG intensity, maintenance therapy, endpoint definitions, use of central pathology, and follow-up duration [17,19,20,21]. The magnitude of this problem is easily underestimated: in the largest multi-institutional gemcitabine–docetaxel series, only 38% of patients met contemporary BCG-unresponsive criteria and 31 had low-grade disease [16,29], and in the long-term Iowa cohort the corresponding proportion was 35% [19]. Supplementary Table S2 sets out, study by study, how closely each cohort corresponds to the strict definition. These differences make indirect comparisons vulnerable and justify a conservative tone.

Furthermore, complete response is an early endpoint that may not capture the full clinical value of a bladder-sparing strategy; durable recurrence-free survival, bladder preservation, and quality of life are equally important [17,18,19]. Cystectomy-free survival should not be interpreted as synonymous with durable disease control, because cystectomy decisions are influenced by surgical fitness, patient preference, physician recommendation, access, and competing risks; accordingly, FDA guidance cautions that cystectomy-related endpoints can be confounded by patient and physician preferences, particularly in single-arm studies [7].

### 9.3. Patient-Reported Outcomes, Treatment Burden, Toxicity, and Cost

Quality of life is a central rationale for considering bladder-sparing therapy, but a retained bladder is not itself a patient-reported outcome. Bladder-sparing therapy may avoid urinary diversion and some early surgical morbidity, yet repeated transurethral resection, intravesical or systemic treatment, cystoscopy, biopsy, and uncertainty about recurrence impose urinary symptoms, sexual and functional effects, anxiety, time burden, and financial toxicity. Conversely, radical cystectomy may produce early functional decline and diversion-related urinary, bowel, sexual, and body-image effects, but may also reduce ongoing treatment burden and recurrence anxiety. Counseling should therefore compare complete treatment pathways rather than presume that anatomical bladder preservation guarantees superior quality of life.

The most direct contemporary strategy-level evidence comes from CISTO, a pragmatic prospective observational cohort of 570 patients with recurrent high-grade NMIBC after prior induction BCG who were candidates for both approaches. Patient-reported physical function was significantly worse in the radical cystectomy arm at 3 months, but by 9 months there was no difference between arms, and at 12 months physical function did not differ (average treatment effect 0.9; 95% CI −0.6 to 2.4; *p* = 0.22). Radical cystectomy was associated with better emotional function, better generic health-related quality of life, lower depression and anxiety, and lower financial burden, whereas bladder-sparing therapy was associated with better bowel and sexual health. Radical cystectomy was also associated with a higher risk of adverse and serious adverse events, including a 90-day mortality rate of 2.5%. Cancer-specific survival at 12 months was 99% with bladder-sparing therapy and 96% with radical cystectomy, a difference that did not reach statistical significance in the study’s weighted analysis [9]. Because treatment was self-selected, the population was broader than strictly defined BCG-unresponsive disease, bladder-sparing regimens were heterogeneous, and follow-up is short, CISTO does not establish causal superiority of either pathway. It does, however, challenge the assumption that bladder preservation uniformly preserves quality of life, and it demonstrates that the trade-off is domain-specific and time-dependent.

Regimen-specific evidence remains incomplete. A patient-reported outcome analysis of N-803 plus BCG reported broadly stable physical function, global health, and NMIBC-specific summary scores during treatment, but the absence of a concurrent comparator and the potential for attrition and responder bias limit interpretation [54]. SunRISe-1 also collected prospective quality-of-life data during TAR-200 treatment, but these too are single-arm and non-comparative [17]. No analogous prospective comparative patient-reported evidence exists in strictly defined BCG-unresponsive cohorts for sequential gemcitabine–docetaxel or gemcitabine monotherapy. This is a fundamental gap in the evidence base rather than a minor omission: the principal justification for bladder-sparing therapy is quality of life, yet quality of life is the outcome least rigorously measured. Future trials and prospective registries should prespecify longitudinal generic and bladder cancer-specific measures encompassing urinary, bowel, sexual, physical, emotional, and social function, body image, treatment time, financial toxicity, fear of recurrence, and decisional regret, analyzed in the intention-to-treat population and after treatment failure and salvage cystectomy so that survivor and responder bias are reduced [55]. Until such evidence matures, quality-of-life superiority should not be asserted for any individual bladder-sparing regimen, or for bladder-sparing therapy over radical cystectomy.

Toxicity profiles also influence selection. Intravesical chemotherapy regimens generally cause local urinary adverse events with low systemic exposure, whereas pembrolizumab introduces the possibility of systemic immune-related toxicity [15,16,20]. Cost-effectiveness evidence in BCG-unresponsive NMIBC is model-based and sensitive to payer perspective, willingness-to-pay threshold, treatment sequencing, and drug or device pricing. Published analyses have generally suggested favorable cost-effectiveness for intravesical gemcitabine–docetaxel under selected assumptions, whereas pembrolizumab, nadofaragene firadenovec, and other higher-cost strategies may be less favorable depending on modeled price and sequencing; these economic findings should not be interpreted as evidence of clinical superiority [56,57,58]. TAR-200 mitigates the short-dwell limitation through sustained intravesical delivery but requires catheter-based insertion, indwelling device management, and scheduled removal, which may affect implementation in routine practice [36].

### 9.4. Molecular Resistance and Biomarker Research Priorities

Mechanistic data should be used to generate clinical hypotheses rather than to rank bladder-sparing therapies. The decline in disease control observed with conventional intravesical gemcitabine is pharmacologically compatible with limited dwell time and urinary washout, whereas TAR-200 was specifically designed to prolong local gemcitabine exposure. Sequential gemcitabine–docetaxel imposes two non-overlapping cytotoxic stresses, but sequence-specific synergy and a clinically meaningful incremental contribution of docetaxel have not been demonstrated in human NMIBC, and a recent retrospective comparison found no oncologic advantage over gemcitabine monotherapy [34]. Reduced gemcitabine sensitivity can plausibly result from impaired nucleoside transport or activation, increased catabolism, ribonucleotide-reductase upregulation, enhanced DNA-damage tolerance, and epithelial–mesenchymal transition or stem-like tumor-cell programs, while docetaxel resistance may involve altered tubulin biology, adenosine triphosphate-binding cassette (ABC) transporter-mediated efflux, and apoptosis escape [14,22]. Experimental work linking the Snai2–NADSYN1–PHB axis to bladder cancer progression and mesenchymal biology provides one worked example of the transition node, although it has not been validated as a predictor of response to any intravesical regimen [59]. Two of these mechanisms generate clinically testable predictions, and it is worth stating explicitly why the evidence reviewed above cannot yet test either of them. First, the exposure-time dependence of gemcitabine predicts that regimens differing only in dwell time should differ in durability. The available data do not demonstrate this: the sustained-release system reported a median duration of response of 25.8 months, but the long-term conventional-instillation gemcitabine–docetaxel cohort reported a median duration of response of 25 months, and the two figures derive from different populations, different phenotype mixes and different response-ascertainment schedules [17,19]. A dwell-time effect can therefore be hypothesized but not inferred, and testing it would require a randomized comparison of delivery format at a fixed drug and schedule. Second, field-change CIS biology, incomplete eradication after transurethral resection, and limited drug contact with occult or flat lesions predict that CIS-containing disease should respond less durably than resected papillary-only disease to the same agent. No study reviewed here provides that within-agent contrast in a form that would test the prediction, because CIS complete-response and duration-of-response endpoints are not interchangeable with papillary-only disease-free survival. Both statements are therefore hypotheses that the present evidence cannot adjudicate, and neither identifies which patient should receive one bladder-sparing regimen rather than another.

The newer bladder-sparing agents exploit complementary immune or virotherapeutic mechanisms: nadofaragene firadenovec induces local interferon alfa-2b production through a non-replicating adenoviral vector [21,46]; nogapendekin alfa inbakicept is an interleukin-15 (IL-15) superagonist that expands and activates natural killer (NK) cells and CD8-positive effector and memory populations alongside BCG [40,44]; and cretostimogene grenadenorepvec achieves Rb/E2F-selective oncolysis with granulocyte–macrophage colony-stimulating factor (GM-CSF)-amplified antigen presentation [47,49,60]. The practical consequence for patient selection is one of modality rather than of molecule: because none of these mechanisms overlaps with nucleoside-analogue or taxane cytotoxicity, failure of intravesical chemotherapy carries no mechanistic expectation of cross-resistance to a gene-based, cytokine-based or oncolytic approach, which supports switching modality rather than repeating the same class in a patient who remains a candidate for further bladder-sparing therapy [12]. This is a biological rationale for sequencing, not evidence of benefit from it, and it does not alter the requirement to reconsider cystectomy at every failure. It should also be noted that the apparent spread in complete response rates across the comparator agents is not, on current evidence, attributable to these mechanistic differences: the studies differ in endpoint timing, response ascertainment, central review and mandatory-biopsy schedules, and a complete response ascertained at any time is not interchangeable with one assessed at a fixed 3-month time point.

Urinary tumor DNA (utDNA) and molecular subtyping are similarly investigational. Detectable baseline or post-treatment utDNA was associated with lower response and shorter recurrence-free or event-free survival in correlative analyses nested within single-arm nadofaragene and atezolizumab studies [61,62], urinary comprehensive genomic profiling has been evaluated for diagnosis and surveillance in a multicenter case–control validation study [63], and transcriptomic NMIBC and BCG-response subtypes carry reproducible prognostic associations [64,65,66,67,68,69,70]. None of these establishes a treatment-by-biomarker interaction, demonstrates superiority of one bladder-sparing regimen over another, validates molecular clearance as a surrogate endpoint, or shows benefit from biomarker-guided switching or cystectomy. These tests should complement—not replace—cystoscopy, cytology and indicated biopsy, and should not enter routine treatment algorithms outside prospective protocols. Throughout, such findings are described as prognostic or correlative; the term predictive is reserved for markers with a prospectively validated treatment-by-biomarker interaction.

Future biomarker studies should prespecify analytically locked assays, cut-offs and sampling time points, formal treatment-by-biomarker interaction tests, and independent validation, and should test whether biomarker-guided escalation, switching or earlier cystectomy improves durable high-grade recurrence-free survival, bladder-intact survival and patient-reported outcomes. The relevant endpoint is demonstrated clinical utility beyond conventional clinicopathologic risk assessment, not biomarker association alone.

### 9.5. Future Directions

Future research should prioritize prospective comparative effectiveness, standardized definitions of BCG exposure and failure, separate reporting of CIS-containing and papillary-only disease, harmonized response-assessment schedules, and longer-term progression and cancer-specific outcomes. Randomized comparisons among bladder-sparing options—though logistically difficult because of patient preferences regarding cystectomy—would clarify whether high early response rates translate into durable bladder preservation and progression avoidance [17]. Carefully designed prospective observational studies, emulated target trials, and standardized real-world registries can reduce, although not eliminate, confounding, and should characterize outcomes in patients under-represented in trials, including those with frailty, major comorbidity, variant histology, upper-tract risk, prostatic urethral involvement, or limited ability to comply with intensive surveillance. Pragmatic trials and registries should incorporate treatment burden and patient-reported outcomes alongside oncologic endpoints, and biomarker studies should be embedded prospectively and considered successful only if they demonstrate incremental clinical utility.

### 9.6. Limitations

This review has several limitations. First, it is a narrative rather than a systematic review and therefore does not provide exhaustive study identification, dual-reviewer screening, formal risk-of-bias assessment, quantitative synthesis, or certainty-of-evidence grading; the electronic search was moreover restricted to PubMed and the Cochrane Library, so records indexed only in Embase or other databases may have been missed, and non-English records were excluded at screening; study selection and emphasis may consequently reflect author judgment, and any apparent differences among therapies may reflect study design, case mix, phenotype, endpoint definition, assessment schedule, or follow-up rather than true comparative effectiveness. Second, the included studies differ in BCG-failure definitions, adequacy and timing of prior BCG, inclusion of CIS versus papillary-only disease, maintenance schedules, endpoint timing, central pathology review, mandatory-biopsy protocols, and follow-up duration. Third, several key therapies are supported by single-arm registration trials or retrospective observational cohorts, limiting causal inference and precluding reliable indirect efficacy rankings. Fourth, complete response, recurrence-free survival, cystectomy-free survival, and bladder preservation are not interchangeable endpoints, and early complete response should not be interpreted as durable oncologic control. Fifth, safety data are reported using different metrics across sources—investigator-attributed treatment-related adverse events, treatment-emergent adverse events, and regulatory adverse reactions—which are not interchangeable and are presented here with their source definitions retained. Sixth, dedicated patient-reported outcome data in strictly defined BCG-unresponsive populations remain limited. Finally, mechanistic and biomarker associations are largely hypothesis-generating, and regulatory status, availability, and prescribing information remain time-sensitive.

## 10. Conclusions

Gemcitabine-based intravesical therapies expand bladder-sparing options for selected patients with confirmed BCG-unresponsive NMIBC who are unfit for or decline radical cystectomy. Gemcitabine monotherapy is active and generally well tolerated but demonstrates declining disease control over time in contemporary high-risk populations. Sequential gemcitabine–docetaxel is supported by substantial but predominantly retrospective real-world experience and should be described as a commonly used off-label, practice-based option; its incremental benefit over gemcitabine alone, and its comparative effectiveness against approved agents or cystectomy, remain unproven. TAR-200/INLEXZO produced a high complete response rate and durable responses in a single-arm phase 2b trial and is the only on-label sustained-delivery option for CIS-containing disease, but randomized comparative effectiveness is not established. Treatment selection should be phenotype- and label-aware and should incorporate surgical fitness, toxicity, treatment burden, access, and domain-specific quality-of-life trade-offs. Any bladder-sparing strategy should be time-limited and surveillance-intensive, with prespecified failure criteria and prompt radical cystectomy for high-grade persistence, recurrence, or progression in surgically fit patients. Biomarker-guided regimen selection remains investigational.

## Figures and Tables

**Table 1:** Evidence summary for gemcitabine-based and comparator bladder-sparing therapies in BCG-unresponsive high-risk NMIBC.

Therapy	Population and Applicability to the Strict BCG-Unresponsive Definition	Evidence Design	Key Efficacy (Endpoint and Time Point)	Standardized Safety (Metric and Denominator)	Principal Limitation
Gemcitabine monotherapy (off-label)	Historical and mixed BCG-failure cohorts (BCG-refractory, BCG-relapsing, BCG-intolerant); contemporary cohorts are study-defined using a 12-month window for all phenotypes. CIS-predominant and papillary disease.	Phase II trials; one small randomized trial versus repeat BCG; Cochrane review; contemporary retrospective real-world series.	MSK (*n* = 30): CR 50% at 8 weeks; median RFS 3.6 months. SWOG S0353 (47 evaluable): 3-month DFS 47%; 1-/2-year RFS 28%/21%. Korean real-world (*n* = 131): 1-/2-year RFS 68%/42%. Single-arm study (*n* = 36): DFS 44.4%/31.7% at 12/24 months.	SWOG S0353, toxicity population *n* = 55 (investigator-graded): grade 3 in 3/55 (5.5%); no grade 4–5; 1/55 discontinued, for a grade 2 event. Grade ≥ 3 TRAE not consistently reported elsewhere (NR); 5.3% of 131 patients did not complete induction in the contemporary Korean series.	Older and mixed definitions; limited durability; heterogeneous schedules; grade-specific toxicity inconsistently reported.
Sequential gemcitabine–docetaxel (off-label)	Predominantly mixed BCG-exposed cohorts—in the largest multi-institutional series only 105/276 (38%) met contemporary criteria and 31 had low-grade disease—plus one strict cohort (*n* = 102) and one restricted comparative cohort (*n* = 95). CIS-containing and papillary disease.	Original description; multi-institutional and long-term retrospective series; one small prospective non-randomized cohort; comparative observational analysis versus additional BCG; retrospective comparative-effectiveness analysis versus gemcitabine; scoping review.	Multi-institutional (mixed, *n* = 276): 1-/2-year HG-RFS 65%/52%. Strict cohort (*n* = 102): 6-/12-/24-month HG-RFS 78%/65%/49%. Iowa (*n* = 97, 35% strictly BCG-unresponsive): 5-year HG-RFS 30%, CFS 75%, CSS 91%. Prospective cohort (*n* = 39 enrolled, 37 evaluable, 72% BCG-unresponsive): 1-year HG-DFS 67% among evaluable patients. Versus gemcitabine monotherapy: no improvement in HG-RFS (adjusted HR 1.09; 95% CI 0.65–1.82).	Prospective cohort *n* = 39 (unattributed adverse events, CTCAE v5.0): any event 30/39 (77%); one grade 3 event (pyelonephritis requiring hospitalization, 2.6%), after which treatment was successfully resumed; discontinuation 3/39 (7.7%). Retrospective comparison (unattributed): grade ≥ 3 events in 2.4% of 123 patients receiving the doublet versus 2.3% of 173 receiving gemcitabine alone. Multi-institutional series: CTCAE grading NR; 9/276 (3.3%) unable to complete induction.	No randomized comparison in strictly defined BCG-unresponsive disease; mixed eligibility; incremental benefit over gemcitabine monotherapy unproven; off-label in both phenotypes.
Gemcitabine intravesical system (INLEXZO; formerly TAR-200) *	US label: BCG-unresponsive NMIBC with CIS, with or without papillary tumors. Trial cohorts applied contemporary BCG-unresponsive eligibility; papillary-only activity is off-label.	Single-arm phase 2b (SunRISe-1); FDA approval and prescribing information.	CIS cohort (*n* = 85): centrally confirmed complete response at any time 82.4%; median DOR 25.8 months. Papillary-only cohort (*n* = 52): 12-month DFS 70.2%.	SunRISe-1 CIS cohort *n* = 85 (TRAE): grade ≥ 3 12.9%; serious 5.9%; discontinuation 3.5%; no treatment-related deaths. Papillary cohort *n* = 52 (TRAE): grade ≥ 3 13.5%; serious 5.8%; discontinuation 7.7%. Label adverse reactions (*n* = 85, different metric): serious 24%; discontinuation 7%; fatal 1.2%.	Single-arm; no comparative data; device insertion and removal logistics with a 3-week indwelling period; label warning on delayed cystectomy (7/83 evaluable patients, 8%, progressed to ≥T2).
Pembrolizumab *	US label: BCG-unresponsive NMIBC with CIS, with or without papillary tumors, in patients ineligible for or electing not to undergo cystectomy. The papillary-only cohort is contextual and off-label.	Single-arm phase 2 (KEYNOTE-057 Cohorts A and B); FDA approval.	Cohort A (CIS, *n* = 101): CR 41% at 3 months; median DOR 16.2 months; 46% of responders durable ≥12 months. Cohort B (papillary-only, *n* = 132): 12-month DFS 43.5%.	Cohort A *n* = 101 (TRAE): grade 3–4 13%; serious 8%; discontinuation 7%; no treatment-related deaths. Cohort B *n* = 132 (TRAE): grade 3–4 14%; serious 13%; no treatment-related deaths.	Systemic immune-related toxicity; no separate papillary-only indication; cohort-specific safety data must not be pooled or transposed.
Nadofaragene firadenovec-vncg *	US label: BCG-unresponsive NMIBC with CIS, with or without high-grade Ta/T1. The pivotal trial excluded lymphovascular invasion, micropapillary histology, prostatic urethral involvement and upper-tract disease.	Single-arm phase 3 with 5-year follow-up; FDA approval summary.	CR 53.4% at 3 months (51% in the FDA review population); 45.5% of responders durable at 12 months; median DOR 9.7 months; bladder preservation in approximately half of patients at 5 years.	Phase 3 safety population *n* = 157 (study-drug-related): grade 3–4 in 6/157 (4%); serious in 3/157 (1.9%); discontinuation in 3/157 (1.9%); no treatment-related deaths. All-cause grade 3–4 was 29/157 (18%).	Single-arm; restrictive eligibility limits generalizability; adenoviral handling requirements and 3-monthly instillation logistics.
Nogapendekin alfa inbakicept-pmln plus BCG *	US label: BCG-unresponsive NMIBC with CIS, with or without papillary tumors. Requires concomitant BCG.	Single-arm (QUILT-3.032); FDA approval summary.	CR 71% (58/82) with median DOR 26.6 months in the primary report; CR 62% in the FDA efficacy population, with 58% and 40% of responders durable ≥12 and ≥24 months.	FDA safety population *n* = 88 (label adverse reactions, not investigator-attributed TRAEs): serious 16%; permanent discontinuation 7%; one fatal adverse reaction (1.1%); aggregate grade ≥ 3 rate NR. The primary publication, which reports treatment-emergent rather than label-defined events for patients receiving BCG plus nogapendekin alfa inbakicept, states that most events were grade 1–2 (86%) and that three grade 3 immune-related treatment-emergent events occurred; it does not report a pooled safety denominator that we were able to verify, so none is given here.	Single-arm; BCG-dependent and therefore shortage-sensitive; the regulatory adverse-reaction metric is not comparable with trial TRAE rates.
Valrubicin (historical)	US label: BCG-refractory CIS in patients for whom immediate cystectomy would carry unacceptable morbidity or mortality. Pre-definition population.	Historical non-comparative approval cohort; FDA label.	Complete response confirmed by biopsy and cytology at 6 months in 16/90 (18%).	Label safety population *n* = 230; grade ≥ 3, serious and fatal event rates NR; 7/143 (5%) of patients scheduled for six instillations did not complete them because of local bladder symptoms.	Historical population; low response rate and durability; not a contemporary preferred option.
Cretostimogene grenadenorepvec (investigational)	BCG-unresponsive CIS; contemporary trial eligibility. No marketing approval as of 4 August 2026.	Single-arm phase 3 (BOND-003 Cohort C).	Centrally confirmed complete response at any time in 83 of 110 evaluable patients (75.5%); estimated 12- and 24-month DOR 64.2% and 60.1%.	BOND-003 Cohort C, safety population *n* = 112 (TRAE): grade 3–4 events 0/112; treatment-related discontinuations 0/112; treatment-related deaths 0/112. Any treatment-related event 71/112 (63%); serious treatment-related events 2/112 (2%), both grade 2.	Investigational; single-arm; not approved, and therefore not an available comparator in routine practice.

* Denotes a current U.S. FDA-approved indication for BCG-unresponsive NMIBC with carcinoma in situ, with or without papillary tumors. No agent listed holds a papillary-only indication; activity reported in papillary-only cohorts should not be interpreted as a separate labeled indication. Safety terminology has been retained from each primary source. TRAE denotes an investigator-attributed treatment-related adverse event; TEAE denotes any event arising or worsening after treatment initiation irrespective of causality; FDA-label adverse reactions are reported using regulatory definitions and may include laboratory abnormalities. Denominators differ between efficacy and safety populations within the same study. Percentages are therefore not directly comparable across rows and NR indicates that a comparable metric was not reported—it does not indicate the absence of events. Efficacy outcomes are likewise not directly comparable. Some studies reported complete response at a fixed 3-month assessment, whereas SunRISe-1 and BOND-003 report complete response at any time; central review procedures, mandatory-biopsy schedules and follow-up duration also differ. CIS complete-response and duration-of-response endpoints are not interchangeable with disease-free or recurrence-free survival after complete resection of papillary-only disease. Abbreviations: BCG, bacillus Calmette–Guérin; CFS, cystectomy-free survival; CI, confidence interval; CIS, carcinoma in situ; CR, complete response; CSS, cancer-specific survival; CTCAE, Common Terminology Criteria for Adverse Events; DFS, disease-free survival; DOR, duration of response; HG-DFS, high-grade disease-free survival; HG-RFS, high-grade recurrence-free survival; HR, hazard ratio; MSK, Memorial Sloan Kettering; NMIBC, non-muscle-invasive bladder cancer; NR, not reported; RFS, recurrence-free survival; TEAE, treatment-emergent adverse event; TRAE, treatment-related adverse event.

## Data Availability

No new data were created or analyzed in this study. Data sharing is not applicable.

## Supplementary Materials

The following supporting information can be downloaded at: https://www.mdpi.com/article/10.3390/biomedicines14081821/s1, Table S1: Full electronic search strings for PubMed and the Cochrane Library, with the record counts returned and the date each database was searched; Table S2: Study-level applicability audit against the contemporary BCG-unresponsive definition.
