# Supplementary material for: Gemcitabine-Based Bladder Preservation in BCG-Unresponsive High-Risk NMIBC: Evidence, Limitations, and Clinical Positioning
Source: Biomedicines. 2026 Aug 13;14(8):1821. doi: 10.3390/biomedicines14081821 (PMC13509982; doi:10.3390/biomedicines14081821)
Supplement: Supplementary file 1 [file biomedicines-14-01821-s001.zip › biomedicines-4453349-supplementary.pdf]

Supplementary Table S1

Full electronic search strategies

Manuscript: “Gemcitabine-Based Bladder Preservation in BCG-Unresponsive High-Risk NMIBC: Evidence, Limitations, and Clinical Positioning”. All searches were executed on 4 August 2026. Two databases were searched: MEDLINE via PubMed and the Cochrane Library. Embase was not available to the authors and was not searched; this restriction is stated in the Methods and in the Limitations of the manuscript. Search lines within a database overlap deliberately and were combined before de-duplication; the record counts below are therefore not additive. This is a targeted narrative review: the strategies are reported for transparency and reproducibility, and no PRISMA flow diagram, duplicate screening, formal risk-of-bias assessment or certainty grading was performed.

A. MEDLINE via PubMed (NLM interface)

Date searched: 4 August 2026 · Date limits applied within each line: 1 January 1990 to 4 August 2026 · No language restriction applied at the search stage; non-English records were excluded at screening.

#	Concept	Search string as executed	Records
#1	Bladder cancer population AND gemcitabine AND intravesical administration	("urinary bladder neoplasms"[MeSH Terms] OR "bladder cancer"[Title/Abstract] OR "bladder neoplasm*"[Title/Abstract] OR "NMIBC"[Title/Abstract] OR "non-muscle-invasive bladder cancer"[Title/Abstract] OR "non muscle invasive bladder cancer"[Title/Abstract] OR "nonmuscle invasive bladder cancer"[Title/Abstract]) AND ("gemcitabine"[MeSH Terms] OR "gemcitabine"[Title/Abstract]) AND ("administration, intravesical"[MeSH Terms] OR "intravesical"[Title/Abstract] OR "instillation*"[Title/Abstract]) AND ("1990/01/01"[Date - Publication] : "2026/08/04"[Date - Publication])	415
#2	Bladder cancer population AND any BCG-failure descriptor	("urinary bladder neoplasms"[MeSH Terms] OR "bladder cancer"[Title/Abstract] OR "bladder neoplasm*"[Title/Abstract] OR "NMIBC"[Title/Abstract] OR "non-muscle-invasive bladder cancer"[Title/Abstract] OR "non muscle invasive bladder cancer"[Title/Abstract] OR "nonmuscle invasive bladder cancer"[Title/Abstract]) AND ("BCG-unresponsive"[Title/Abstract] OR "BCG unresponsive"[Title/Abstract] OR "BCG-refractory"[Title/Abstract] OR "BCG refractory"[Title/Abstract] OR "BCG-relapsing"[Title/Abstract] OR "BCG relapsing"[Title/Abstract] OR "BCG failure"[Title/Abstract] OR "BCG-exposed"[Title/Abstract] OR "BCG intolerant"[Title/Abstract]) AND ("1990/01/01"[Date - Publication] : "2026/08/04"[Date - Publication])	621
#3	Sequential gemcitabine–docetaxel	("gemcitabine"[Title/Abstract] AND "docetaxel"[Title/Abstract]) AND ("intravesical"[Title/Abstract] OR "bladder"[Title/Abstract]) AND ("urinary bladder neoplasms"[MeSH Terms] OR "bladder cancer"[Title/Abstract] OR "bladder neoplasm*"[Title/Abstract] OR "NMIBC"[Title/Abstract] OR "non-muscle-invasive bladder cancer"[Title/Abstract] OR "non muscle invasive bladder cancer"[Title/Abstract] OR "nonmuscle invasive bladder cancer"[Title/Abstract]) AND ("1990/01/01"[Date - Publication] : "2026/08/04"[Date - Publication])	157
#4	Sustained-release gemcitabine intravesical system	("urinary bladder neoplasms"[MeSH Terms] OR "bladder cancer"[Title/Abstract] OR "bladder neoplasm*"[Title/Abstract] OR "NMIBC"[Title/Abstract] OR "non-muscle-invasive bladder cancer"[Title/Abstract] OR "non muscle invasive bladder cancer"[Title/Abstract] OR "nonmuscle invasive bladder cancer"[Title/Abstract]) AND ("TAR-200"[Title/Abstract] OR "Inlexzo"[Title/Abstract] OR "gemcitabine intravesical system"[Title/Abstract] OR "gemcitabine releasing intravesical system"[Title/Abstract] OR "SunRISe"[Title/Abstract] OR "intravesical drug-releasing system"[Title/Abstract]) AND ("1990/01/01"[Date - Publication] : "2026/08/04"[Date - Publication])	35
#5	Comparator and investigational bladder-sparing agents in BCG-failure disease	("urinary bladder neoplasms"[MeSH Terms] OR "bladder cancer"[Title/Abstract] OR "bladder neoplasm*"[Title/Abstract] OR "NMIBC"[Title/Abstract] OR "non-muscle-invasive bladder cancer"[Title/Abstract] OR "non muscle invasive bladder cancer"[Title/Abstract] OR "nonmuscle invasive bladder cancer"[Title/Abstract]) AND ("BCG-unresponsive"[Title/Abstract] OR "BCG unresponsive"[Title/Abstract] OR "BCG-refractory"[Title/Abstract] OR "BCG refractory"[Title/Abstract] OR "BCG-relapsing"[Title/Abstract] OR "BCG relapsing"[Title/Abstract] OR "BCG failure"[Title/Abstract] OR "BCG-exposed"[Title/Abstract] OR "BCG intolerant"[Title/Abstract]) AND ("pembrolizumab"[Title/Abstract] OR "nadofaragene firadenovec"[Title/Abstract] OR "Adstiladrin"[Title/Abstract] OR "N-803"[Title/Abstract] OR "nogapendekin"[Title/Abstract] OR "Anktiva"[Title/Abstract] OR "cretostimogene"[Title/Abstract] OR "CG0070"[Title/Abstract] OR "detalimogene"[Title/Abstract] OR "EG-70"[Title/Abstract] OR "valrubicin"[Title/Abstract] OR "atezolizumab"[Title/Abstract] OR "sasanlimab"[Title/Abstract]) AND ("1990/01/01"[Date - Publication] : "2026/08/04"[Date - Publication])	150
#6	Bladder preservation versus cystectomy AND quality of life / patient-reported outcomes	("urinary bladder neoplasms"[MeSH Terms] OR "bladder cancer"[Title/Abstract] OR "bladder neoplasm*"[Title/Abstract] OR "NMIBC"[Title/Abstract] OR "non-muscle-invasive bladder cancer"[Title/Abstract] OR "non muscle invasive bladder cancer"[Title/Abstract] OR "nonmuscle invasive bladder cancer"[Title/Abstract]) AND ("bladder-sparing"[Title/Abstract] OR "bladder sparing"[Title/Abstract] OR "bladder preservation"[Title/Abstract] OR "bladder-preserving"[Title/Abstract] OR	987

#	Concept	Search string as executed	Records
		`"radical cystectomy"[Title/Abstract]) AND ("quality of life"[MeSH Terms] OR "quality of life"[Title/Abstract] OR "patient-reported outcome*"[Title/Abstract] OR "patient reported outcome*"[Title/Abstract]) AND ("1990/01/01"[Date - Publication] : "2026/08/04"[Date - Publication])`	

Records retrieved across PubMed lines #1–#6 before removal of duplicates: 2,365.

B. Cochrane Library (Wiley interface; CDSR and CENTRAL)

Date searched: 4 August 2026 · Issue searched: Issue 8 of 12, August 2026 · Field: Title, Abstract, Keyword. Counts are reported by segment because the interface returns them separately.

#	Concept	Search string as executed	Records (by segment)
#1	Bladder cancer AND gemcitabine AND intravesical administration	`("bladder cancer" OR NMIBC OR "non-muscle-invasive bladder cancer") AND gemcitabine AND (intravesical OR instillation)`	Cochrane Reviews 1; Protocols 0; Trials 172; Editorials 0; Special Collections 0; Clinical Answers 1 — total 174
#2	Bladder cancer AND any BCG-failure descriptor	`(NMIBC OR "bladder cancer" OR "non-muscle-invasive bladder cancer") AND ("BCG-unresponsive" OR "BCG unresponsive" OR "BCG-refractory" OR "BCG refractory" OR "BCG failure" OR "BCG relapsing")`	Cochrane Reviews 1; Protocols 0; Trials 122; Editorials 0; Special Collections 0; Clinical Answers 0 — total 123
#3	Sequential gemcitabine–docetaxel	`gemcitabine AND docetaxel AND bladder AND (intravesical OR instillation)`	Cochrane Reviews 0; Protocols 0; Trials 20; Editorials 0; Special Collections 0; Clinical Answers 0 — total 20

Records retrieved across Cochrane lines #1–#3 before removal of duplicates: 317.

C. Sources not captured by bibliographic databases

Source	What was checked	Date checked
**Guideline documents**	European Association of Urology Non-muscle-invasive Bladder Cancer Guideline (2024 summary and 2026 edition); AUA/SUO Non-Muscle Invasive Bladder Cancer Guideline 2024 Amendment; International Bladder Cancer Group consensus statement and bladder-sparing recommendations.	4 August 2026
**U.S. FDA regulatory sources**	Drugs@FDA prescribing information for INLEXZO (gemcitabine intravesical system), ADSTILADRIN, ANKTIVA, KEYTRUDA and VALSTAR; FDA drug-approval announcements; FDA draft guidance for industry on BCG-unresponsive NMIBC.	4 August 2026
**Congress proceedings**	American Urological Association, European Association of Urology, Society of Urologic Oncology and ASCO Genitourinary Cancers Symposium abstracts. Used only to identify emerging studies and to confirm publication status. Where a conference figure had been superseded by a peer-reviewed publication, the published value was used and the conference value discarded.	4 August 2026
**Reference lists**	Manual screening of the reference lists of all included primary studies, guidelines and recent reviews.	4 August 2026
**Publication-status verification**	Each cited record was verified against Crossref and the publisher record for authors, title, journal, year, volume, pages and DOI; every quantitative value reported in the manuscript was checked against the primary publication or the current regulatory label.	4 August 2026

Note on interface behavior

PubMed applies automatic term mapping to unquoted or hyphenated terms. An initial version of PubMed line #4 that searched the device identifiers without the bladder-cancer population block returned 1,404 records, because the platform decomposed the hyphenated product identifier into its component terms. The line was therefore constrained by the population block, as reported above, which returned 35 records. This is documented here because the discrepancy would otherwise be irreproducible.

Supplementary Table S2

Applicability of the reviewed studies to the contemporary BCG-unresponsive definition

Categories follow the current EAU/FDA-aligned criteria: BCG-unresponsive includes persistent or recurrent CIS within 12 months or high-grade Ta/T1 recurrence within 6 months after adequate BCG, as well as high-grade T1 disease at the first evaluation after induction BCG. Adequate BCG comprises at least five of six initial-induction doses plus either at least two of six second-induction doses or at least two of three maintenance doses. BCG-exposed includes Ta high-grade and/or CIS at the three-month evaluation after induction BCG only, and delayed high-grade recurrence after inadequate or adequate BCG outside the BCG-unresponsive windows but within 24 months; BCG intolerance is a separate state, and recurrence beyond 24 months is late relapse.

Study (first author, year)	Reported population and eligibility	Applicability to the strict contemporary definition	How it is presented in this review
**Dalbagni, 2006**	Titled BCG-refractory, but eligibility also permitted BCG-intolerant patients (n = 30).	No — historical, pre-definition	Historical mixed cohort; not labeled BCG-unresponsive.
**Skinner (SWOG S0353), 2013**	Recurrence after ≥2 prior BCG courses; last BCG permitted up to 3 years before registration (58 enrolled; 47 evaluable for response; 55 evaluable for toxicity).	Mixed — adequate exposure partly captured, timing not captured	Reported with all three denominators stated explicitly.
**Di Lorenzo, 2010**	Failure after a single BCG induction course.	Indeterminate/mixed — strict criteria not fully reported	Presented as an induction-only mixed cohort; adequate BCG and the high-grade T1 exception cannot be resolved from the aggregate report.
**Hurle, 2020**	BCG-unresponsive or BCG-intolerant (n = 36).	Mixed	Presented as a mixed salvage cohort.
**Lee, 2026**	Adequate BCG; high-grade recurrence within 1 year applied to all phenotypes (n = 131, nine centers).	Partial — study-defined contemporary cohort	Concordance with the FDA 6-month papillary window is explicitly noted as unconfirmed.
**De Jesus Escano, 2025**	n = 127; 57% met the historical BCG-refractory definition and 33% met BCG-unresponsive criteria using a 12-month cut-off.	Subgroup only	Overall estimates are not generalized to the strict population.
**Steinberg, 2015**	Early single-institution experience including BCG-naïve, BCG-intolerant and single-course failures (n = 45).	No — historical case mix	Presented as the foundational dosing report.
**Steinberg, 2020**	n = 276; 105 (38%) met contemporary BCG-unresponsive criteria; 31 patients had low-grade tumors.	Subgroup only	Overall 1- and 2-year estimates are described as mixed-population estimates.
**Chevuru, 2023**	n = 97; 71% CIS-containing; approximately 35% met the BCG-unresponsive definition.	Subgroup only	The strict proportion is stated alongside the 5-year outcomes.
**Yim, 2023**	n = 102, restricted to BCG-unresponsive disease.	Yes — strict cohort	Used as the principal strict-cohort estimate for gemcitabine–docetaxel.
**Taylor, 2025 (versus additional BCG)**	FDA-defined BCG-unresponsive disease (gemcitabine–docetaxel n = 95; additional BCG n = 204).	Yes — strict cohort	Presented as a retrospective comparative association, not causal superiority.
**Pijpers, 2025**	n = 39 enrolled, 37 evaluable for efficacy; 28/39 (72%) BCG-unresponsive; 31 (79%) with CIS.	Mixed overall; strict subgroup reported	Both the overall and the BCG-unresponsive subgroup results are given.
**Scilipoti (EuroGemDoce), 2025**	Completed BCG induction and ≥1 follow-up evaluation required; 95 patients collected at 12 centers, outcomes reported for 75. No adequacy or timing criterion.	Mixed/indeterminate — no adequacy or timing criterion applied	Presented as mixed post-BCG evidence; not labeled strictly BCG-unresponsive.
**Scilipoti, 2026 (failure patterns)**	n = 143 stratified as early non-refractory, early refractory and late BCG-relapsing.	Mixed phenotypes	Used for failure-pattern heterogeneity only. The published report does not resolve the adequacy and timing of prior BCG within the late-relapsing group, so that subgroup is read as non-BCG-unresponsive evidence rather than assigned to a single category.
**Moyer, 2026**	n = 296 overall (gemcitabine 173, gemcitabine–docetaxel 123); BCG-	Subgroup only	The primary comparison is described as broader high-risk NMIBC; the

Study (first author, year)	Reported population and eligibility	Applicability to the strict contemporary definition	How it is presented in this review
	unresponsive sensitivity analysis n = 89; BCG-unresponsive CIS subgroup n = 49.		two subgroup sizes are reported separately.
**Daneshmand (SunRISe-1), 2025**	Contemporary BCG-unresponsive eligibility; CIS cohort n = 85 and papillary-only cohort n = 52.	Yes — strict cohort	Single-arm phase 2b; cohorts reported separately.
**Balar / Necchi (KEYNOTE-057), 2021 / 2024**	Contemporary BCG-unresponsive eligibility; Cohort A (CIS) n = 101, Cohort B (papillary-only) n = 132.	Yes — strict cohort	Cohort-specific efficacy and safety are never combined.
**Boorjian (nadofaragene), 2021**	BCG-unresponsive CIS ± high-grade Ta/T1; excluded lymphovascular invasion, micropapillary histology, prostatic urethral and upper-tract disease.	Yes — strict but selectively enrolled	Eligibility restrictions are stated when generalizing.
**Chamie (QUILT-3.032), 2023**	BCG-unresponsive CIS ± papillary disease; efficacy n = 82, safety n = 88.	Yes — strict cohort	Efficacy and safety denominators are reported separately and are never combined; the regulatory efficacy population used in the FDA review is a further, separately defined denominator.
**Tyson (BOND-003 Cohort C), 2026**	BCG-unresponsive CIS; 110 evaluable patients.	Yes — strict cohort	Presented as investigational, single-arm evidence.
**Howard, 2022**	FDA-defined BCG-unresponsive cohort undergoing cystectomy after 0, 1 or ≥2 lines of bladder-sparing therapy.	Yes — strict cohort	Used only for adverse pathology at delayed cystectomy; subgroup sizes are small and the comparison is non-randomized.
**Taylor, 2025 (bladder-sparing vs upfront cystectomy)**	578 patients meeting FDA BCG-unresponsive criteria at 10 international sites; 28% upfront cystectomy, 72% bladder-sparing therapy; median follow-up 50 months.	Yes — strict cohort	Percentages for recurrence, progression and cystectomy are stated as relative to the bladder-sparing group, not the full cohort.
**Gore (CISTO), 2026**	570 patients with recurrent high-grade NMIBC after prior induction BCG who were candidates for both cystectomy and bladder-sparing therapy; treatment self-selected.	Mixed — broader than the strict definition	Presented as the best available comparative patient-reported outcome evidence. Its 12-month cancer-specific survival figures are quoted descriptively; because treatment was self-selected, they are not presented as a comparative oncologic result.
**Black (SWOG S1605), 2023**	BCG-unresponsive high-risk NMIBC; single-arm phase 2 of intravenous atezolizumab.	Yes — strict cohort	Cited as context only; the agent is not approved and the trial missed its efficacy threshold.
**Steinberg (valrubicin), 2000**	BCG-refractory carcinoma in situ; pre-definition regulatory cohort of 90 patients.	No — historical, pre-definition	Presented as a historical comparator with limited durability.

Applicability reflects the reported BCG exposure, timing and phenotype criteria of each study. Overall estimates from mixed or historical cohorts should not be generalized to a strictly defined contemporary BCG-unresponsive population. This audit describes clinical applicability and does not constitute a formal risk-of-bias or certainty-of-evidence assessment.
